# Cast Porosity Prediction by Means of Thercast Finite Element Analysis

**DOI:** 10.3390/ma19173563

**Published:** 2026-08-22

**Authors:** Serhii Fedoriachenko, Viktoriia Kozechko, Kirill Ziborov, Oleksandr Shvets, Vadim Korol, Valentyn Kozechko, Bartłomiej Jeż

**Affiliations:** 1 Department of Generative Design, Faculty of Mechanical Engineering, Dnipro University of Technology, 49005 Dnipro, Ukraine; fedoriachenko.s.o@nmu.one (S.F.); korol.v.m@nmu.one (V.K.); kozechko.v.i@nmu.one (V.K.); 2 Department of Civil Engineering, Faculty of Civil Engineering, Czestochowa University of Technology, 42-201 Czestochowa, Poland; viktoriia.kozechko@pcz.pl; 3 Dnipro Metallurgical Institute, Ukrainian State University of Science and Technologies, 49010 Dnipro, Ukraine; a_shvets@ua.fm; 4 Department of Mechanics and Fundamentals of Machinery Design, Faculty of Mechanical Engineering, Czestochowa University of Technology, 42-201 Czestochowa, Poland; bartlomiej.jez@pcz.pl

**Keywords:** steel cast, Thercast, porosity, quality, mechanical strength, Niyama criterion

## Abstract

**Highlights:**

- The classical Niyama criterion was supplemented by solid-fraction weighting, while alloy-dependent thermophysical effects were included through the THERCAST model.
- A combined numerical–experimental approach improved shrinkage porosity prediction in steel ingots.
- Optimized cooling conditions reduced the extent of the simulated porosity-prone region in the investigated casting configuration.

**Abstract:**

This research aims to investigate and improve the accuracy of porosity prediction in steel ingot casting by leveraging Thercast finite element simulations. In particular, the study refines the Niyama criterion through additional physical parameters and solidification modeling, aiming to reduce shrinkage porosity and enhance the overall mechanical reliability of cast components. Simulation results reveal that a lower thermal conductivity and faster cooling rates exacerbate shrinkage porosity, while a refined Niyama indicator using explicit solid-fraction weighting, with viscosity, alloy composition, and shrinkage accounted for through the underlying THERCAST material model, improves spatial localization of porosity-prone regions in the investigated case. For the investigated configuration, reducing the cooling rate to around 1.25 K/s decreased the extent of the simulated region classified as porosity-prone relative to the reference case. Furthermore, the analytical porosity–strength relation indicates a material-dependent reduction in strength when the porosity fraction exceeds 2%, underscoring the structural significance of internal voids. This study extends the practical interpretation of the classical Niyama criterion by combining solid-fraction weighting with material-dependent thermophysical inputs, addressing gaps in existing shrinkage porosity models. The approach integrates simulation findings with actual casting defects identified through ultrasonic scanning and metallographic analysis. By merging experimental insights with advanced finite element simulations, foundries can better regulate casting conditions, particularly cooling rates and thermal gradients, to minimize porosity. The refined porosity prediction framework aids in process optimization, improved material utilization, and superior quality assurance of steel ingots.

## 1. Introduction

Heavy ingots serve as critical semi-finished products in the metallurgical industry, particularly in the production of high-quality forgings for mechanical engineering applications. These include crankshafts for ship engines and specialized components for both conventional and nuclear power plants, such as turbines, heat exchangers, and steam generators [1,2,3]. However, the manufacturing process presents challenges, including the segregation of alloying elements, porosity, and structural inconsistencies that can impact mechanical properties and quality control. To meet increasing demands for high-performance steel products, steel manufacturers continuously refine their production technologies. This includes optimizing melting processes, adjusting casting parameters, and improving cooling techniques to enhance material properties and reduce defects.

Steel casting is a fundamental manufacturing process utilized across various industries, including automotive, aerospace, and construction [4]. One of the primary challenges in steel casting is the formation of micro-porosity, which significantly affects the structural integrity and performance of the final product. The necessity of researching micro-porosity in steel casting has led to the adoption of numerical simulation methods, which provide a deeper understanding of porosity formation mechanisms and their impact on product quality.

Micro-porosity is a critical solidification defect in cast metals, particularly in steel and aluminum alloys, that arises due to inadequate feeding of interdendritic regions during the final stages of solidification. These voids, typically on the scale of a few microns to tens of microns, are challenging to detect and predict, yet they significantly impact mechanical properties such as fatigue resistance, tensile strength, and ductility [5,6].

The characterization of micro-porosity involves both qualitative and quantitative approaches. Qualitatively, optical microscopy (OM) and scanning electron microscopy (SEM) enable the visualization of pore morphology, shape, and distribution within the microstructure. These methods provide contextual insights, revealing whether pores are intergranular or intragranular, and whether they align with dendrite arms or are clustered near inclusions or grain boundaries [7,8,9].

Quantitative assessment methods include X-ray computed tomography (XCT) and image-based morphometry, which provide 3D visualization and precise measurement of porosity volume fraction, pore size distribution, and spatial connectivity [10]. XCT, in particular, allows for the non-destructive inspection of casting interiors with resolutions below 5 µm, making it a powerful tool in both research and industrial defect evaluation. Automated image analysis software (e.g., ImageJ 1.54, Dragonfly 3D World 2025) further facilitates statistical quantification of porosity in large image datasets, supporting the calibration of simulation models.

The development of micro-porosity is inherently linked to solidification dynamics, alloy composition, and thermal gradients. During the final stages of freezing, solidifying dendrites restrict liquid flow, particularly in areas where the solid fraction exceeds 0.8–0.9, impeding feeding and gas escape [6]. In high-alloy steels or Al-Si alloys, the presence of eutectic pockets exacerbates this effect. Constitutional supercooling and non-uniform grain growth lead to dendritic structures with varying permeability, affecting the formation and entrapment of micro-voids. Microstructure evolution models based on phase-field simulation and solidification thermodynamics can predict the likelihood of micro-porosity formation. Such models incorporate parameters like cooling rate, solidification time, and primary dendrite arm spacing (PDAS). Recent developments in machine learning further enable CNN-based microstructure prediction from SEM or OM images, which can be used to infer defect-prone regions by identifying features like coarse grains, sharp dendrite tips, or eutectic segregation. Numerical tools such as Thercast NxT 3.0, ProCAST 2025.0, or ANSYS 19.2 Fluent simulate thermal gradients, solidification fronts, and feeding flow during casting. These simulations incorporate porosity prediction criteria, the most notable being the Niyama criterion, which correlates thermal gradient and cooling rate to porosity susceptibility [11].

**Figure 1:**
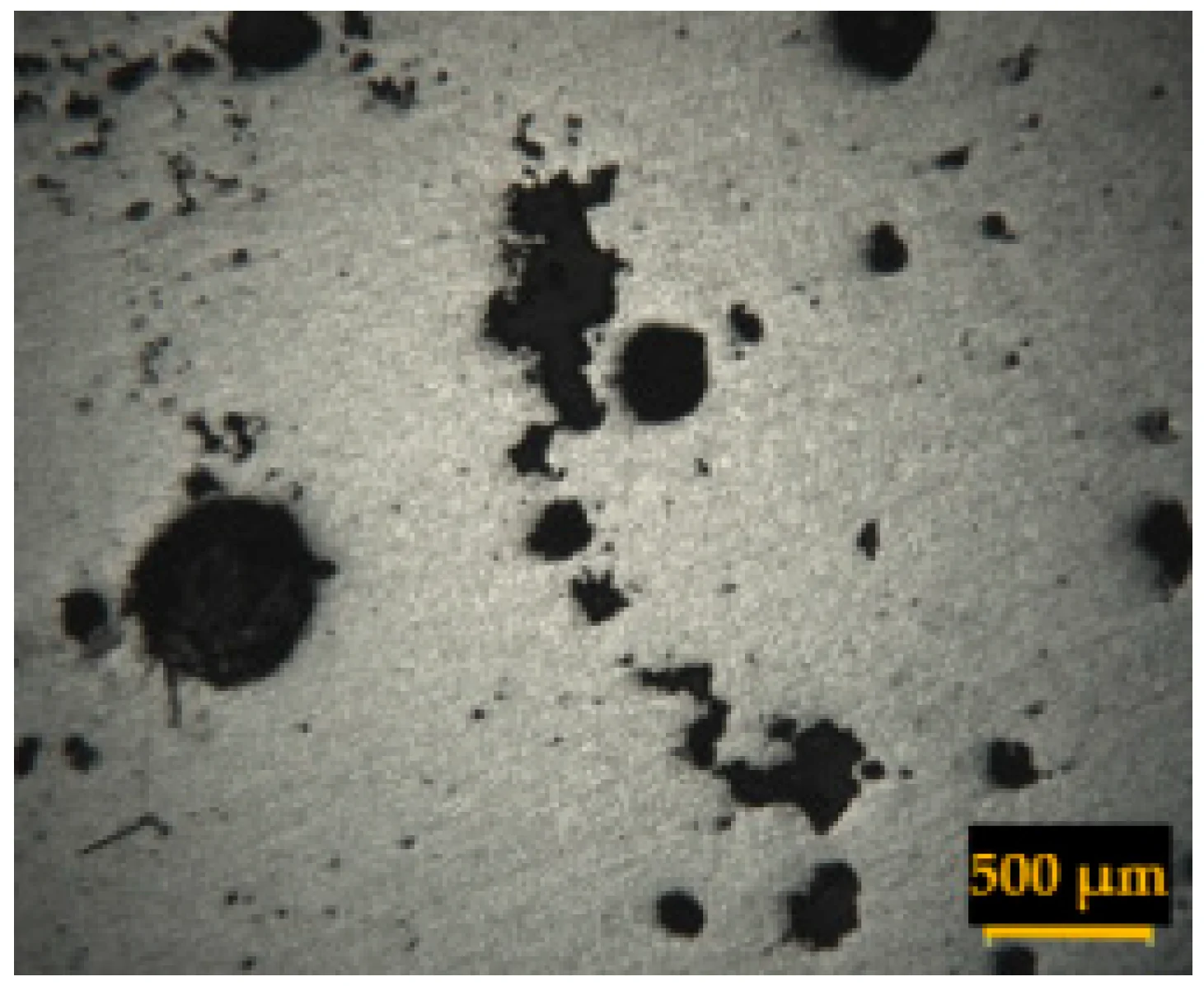
Literature-derived micrograph of gas porosity [14]; not an experimental result of this study.

Micro-porosity (Figure 1) is typically characterized using both qualitative and quantitative measurement techniques. Qualitative methods, such as optical and scanning electron microscopy, provide insights into the morphology and distribution of porosity, while quantitative approaches, including X-ray computed tomography and image analysis, offer precise measurements of porosity volume fraction and size distribution. The ability to accurately measure and analyze porosity is crucial in predicting and mitigating defects in steel casting.

The Niyama criterion [12] is a widely utilized parameter in casting simulations to predict the likelihood of shrinkage porosity in steel castings. It is defined as the local thermal gradient divided by the square root of the local cooling rate. This criterion helps identify regions within a casting that are prone to porosity due to insufficient feeding during solidification [13].

In the context of steel casting, the porosity criterion plays a crucial role in ensuring the integrity and quality of the final product. Stainless steels, known for their corrosion resistance and mechanical properties, require precise control during casting to prevent defects such as shrinkage porosity.

The study in [15] presents a comprehensive examination of the formation, characterization, and influence of porosity and cracks in additively manufactured (AM) tooling alloys. The authors explore both laser powder bed fusion (LPBF) and directed energy deposition (DED) methods, discussing how process parameters such as laser power, scan speed, and layer thickness affect the microstructure of AM parts. A particular emphasis is placed on the influence of thermal gradients and melt pool dynamics, which govern the nucleation of defects like lack-of-fusion pores and keyhole-induced voids. This result is highly relevant for understanding how metallurgical defects in AM processes can be minimized to achieve structural reliability.

In terms of mechanical performance, this outlines how porosity and microcracks drastically reduce fatigue life, ductility, and fracture toughness. The authors link defect morphology, such as spherical gas pores versus irregular lack-of-fusion voids, to their differing effects on failure mechanisms. They also highlight the anisotropic behavior in AM alloys caused by columnar grain growth, which is further exacerbated by defect alignment along build directions. Importantly, the paper includes quantitative comparisons of mechanical properties between conventional and AM alloys, showing that properly optimized AM components can achieve comparable or even superior strength when porosity is controlled.

Lekakh [16] systematically investigated the relationship between metallurgical parameters and micro-porosity formation in ductile iron castings, providing key quantitative results that inform casting design and quality control. The study demonstrated that lower nodularity and increased carbon equivalent (CE) were strongly associated with increased micro-porosity volume fractions. Specifically, castings with CE values near the eutectic composition showed significantly reduced porosity due to improved solidification balance. Furthermore, the research revealed that magnesium fading and insufficient inoculation reduced the number of graphite nodules, thereby promoting porosity formation by altering the solidification morphology and feeding conditions.

Another critical outcome of the paper was the correlation between nodule count and shrinkage porosity severity. The results showed that castings with higher nodule counts exhibited lower porosity, even when produced under similar cooling conditions. This finding reinforces the importance of inoculation and spheroidization control in achieving dense, defect-free structures. Additionally, the author noted that the geometry of the test casting influenced porosity localization, with isolated thermal centers more susceptible to porosity development. This observation supports the integration of thermal and metallurgical modeling for improved casting process optimization.

Lastly, the paper emphasized the role of secondary phases, such as carbides, in increasing porosity risk under fast cooling conditions or improper alloy chemistry. These findings were supported by metallographic image analysis and porosity quantification, offering a detailed understanding of microstructure–defect interactions. The paper concluded with practical recommendations for minimizing porosity through CE adjustment, magnesium treatment control, and optimized thermal design—contributing valuable process–structure–property insights for the ductile iron casting industry.

By applying the Niyama criterion in simulation software, foundries can predict and mitigate these defects, leading to improved casting soundness [15].

Research conducted by Carlson and Beckermann has demonstrated the application of the Niyama criterion in predicting shrinkage-related defects in high-nickel steel and nickel-based alloy castings. Their findings indicate that maintaining Niyama values above a certain threshold is essential to prevent porosity formation [16,17].

Furthermore, they assessed variations in casting simulation predictions using the Niyama criterion. The study highlighted the importance of accurate thermal modeling and the need for standardized approaches in simulation practices to ensure consistent and reliable predictions across different foundries.

In European research, the Niyama criterion has been extensively studied to enhance casting quality. For instance, a study [18] proposed a methodology to define the Niyama criterion reinforced with a solid fraction parameter. This approach aimed to improve the prediction accuracy of porosity defects in casting processes.

The presence of micro-porosity adversely influences the mechanical properties of steel castings. Increased porosity can lead to decreased density, reduced load-bearing capacity, and enhanced susceptibility to crack propagation. Additionally, the interaction between porosity and cracks further compromises the structural strength, leading to premature failure under mechanical stress. Understanding these relationships is essential for improving casting processes and ensuring the production of high-quality components [19].

To address these challenges, advanced numerical simulation tools, such as Thercast, have been developed to model and predict porosity formation. Thercast provides comprehensive simulations that incorporate solidification dynamics, thermal gradients, and shrinkage effects, facilitating accurate predictions of porosity distribution. Specifically, the application of Thercast in Niyama criterion-based evaluations enhances the ability to forecast regions prone to porosity formation, allowing for process optimization and defect minimization.

The integration of numerical methods in steel casting research is indispensable for enhancing product reliability and efficiency. By leveraging tools like Thercast, casting parameters can be optimized, reducing defects and ultimately improving the mechanical performance of steel components. This research aims to investigate the micro-porosity predication in steel casting, its measurement techniques, and implications for structural integrity, while highlighting the importance of numerical simulations in predicting and mitigating casting defects [20].

It is necessary to focus on identifying and characterizing micro-porosity through a combination of qualitative and quantitative measurement techniques. Understanding the influence of micro-porosity and cracks on mechanical strength is a key objective, requiring both experimental data and numerical models to assess their impact. Additionally, the study aims to validate and optimize porosity prediction models by integrating the Thercast simulation tool and the Niyama criterion, which will enhance defect forecasting and reduction strategies in steel casting.

The scientific novelty of this research lies in improving the accuracy of numerical simulations by refining Thercast-based modeling of porosity formation. A significant contribution is the development of a deeper correlation between micro-porosity characteristics and mechanical failure mechanisms, providing new insights into defect-driven degradation. Furthermore, establishing an optimized methodology for predicting and mitigating porosity in steel casting will contribute to higher product quality and structural reliability [21].

This research aims to investigate and improve the accuracy of porosity prediction in steel ingot casting by leveraging Thercast finite element simulations. In particular, the study refines the Niyama criterion through additional physical parameters and solidification modeling, aiming to reduce shrinkage porosity and enhance the overall mechanical reliability of cast components.

## 2. Materials and Methods

The Niyama criterion is a widely used mathematical approach for predicting porosity formation in steel castings. It is defined by the equation below.

Shrinkage porosity is one of the most critical defects in steel casting, as it directly affects mechanical properties such as tensile strength, fatigue resistance, and corrosion behavior.

The Niyama criterion evaluates the balance between the thermal gradient (G) and the cooling rate (dT/dt) during solidification. This study presents an extended mathematical analysis of the Niyama model, its finite element method (FEM) simulation, and its application in metallurgical production by means of numerical simulation [20].

The Niyama number (N_y_) is defined as:

(1) $$N_{y}=\frac{G}{\sqrt{dT/dt}},$$

where G—thermal gradient, °C/mm; dT/dt—cooling rate, °C/s.

A lower Niyama value generally indicates a higher probability of porosity formation due to insufficient feeding and solidification shrinkage.

To analyze the impact of different variables on porosity formation, researchers have studied the change in the thermal gradient and cooling rate influence, and the reliability of the criterion. A key factor affecting porosity prediction is the accuracy of heat transfer models in numerical simulations. Errors in estimating thermal properties, such as thermal conductivity and latent heat of fusion, can lead to incorrect porosity predictions [22].

The THERCAST solution includes the effects of alloy composition, temperature-dependent thermophysical properties, melt viscosity, and solidification shrinkage on the calculated thermal and phase fields. These quantities therefore influence G, |dT/dt|, and fs indirectly. The explicit post-processing modification introduced in this study is the solid-fraction weighting of the classical Niyama criterion, consistent with the combined use of Niyama and solid-fraction analysis [18]:

(2) $$N_{y,mod}=\frac{G}{\sqrt{R}}\sqrt{1-f_{s}}$$

$$R=-\frac{dT}{dt}>0$$

where f_s_ is the local solid fraction (dimensionless) at the selected stage of solidification; (1 − f_s_) is the remaining liquid fraction available for interdendritic feeding.

As the solid fraction approaches unity, the connected liquid fraction decreases and the weighted indicator approaches zero; low N_y,mod_ values therefore identify regions in which both the thermal feeding conditions and the remaining liquid fraction are unfavorable.

The modified indicator is used to rank porosity susceptibility spatially; it is not interpreted as a direct equation for porosity volume fraction.

The enhanced indicator was evaluated by comparing the simulated defect-prone region with industrial ultrasonic inspection and subsequent sectioning of the indicated casting area. X-ray computed tomography is identified only as a recommended method for future three-dimensional quantitative validation and was not used to generate the results reported here.

The conventional and modified Niyama distributions were compared graphically. Owing to the industrial case-study character of the retained dataset, the present comparison emphasizes spatial localization; a full MAE/RMSE and probabilistic sensitivity analysis is reserved for a future registered volumetric dataset.

**Figure 2:**
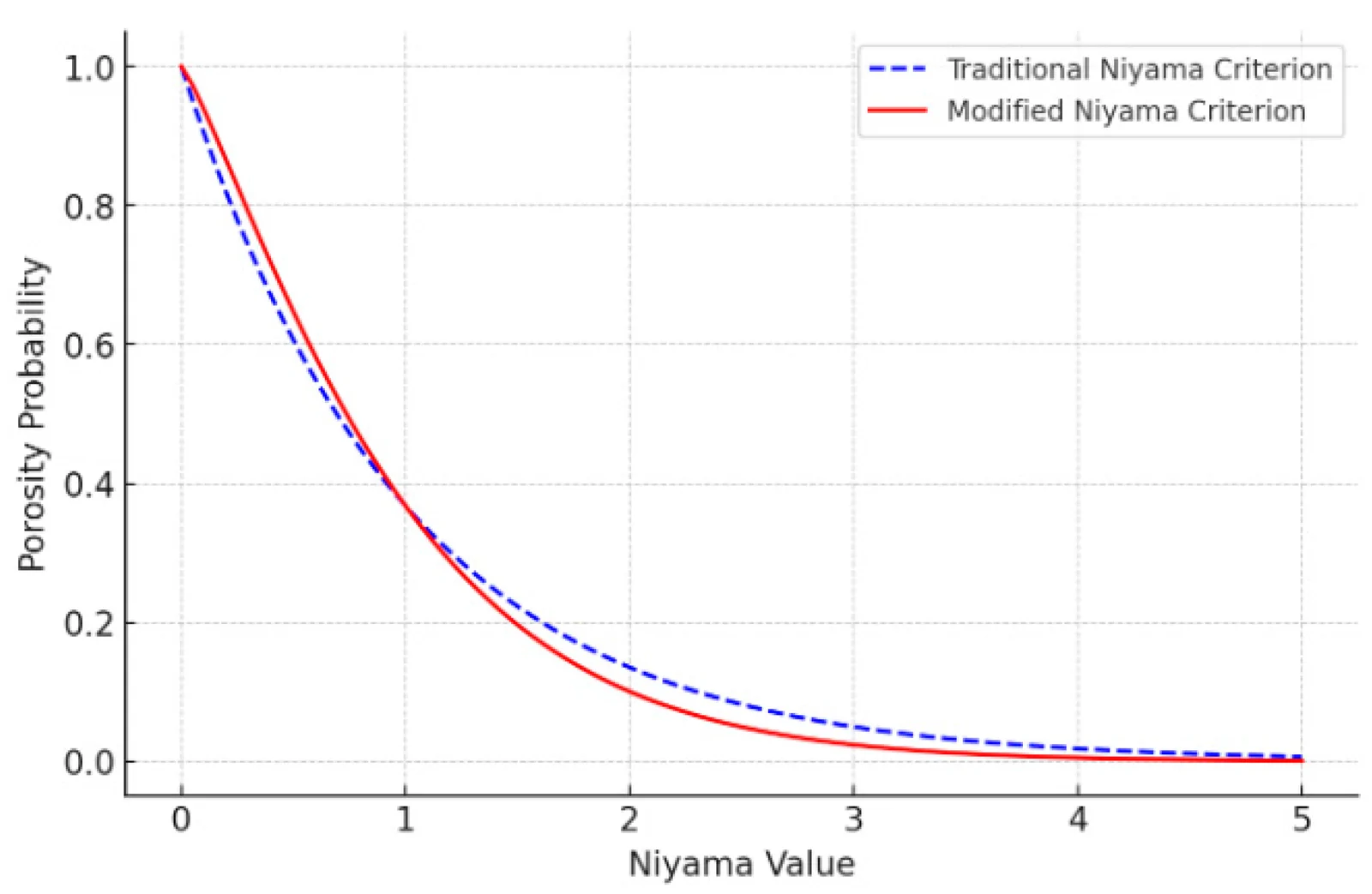
Schematic qualitative comparison of porosity-risk trends for the conventional and modified Niyama criteria.

Figure 2 provides a schematic qualitative comparison of the porosity-risk response for the conventional and modified Niyama criteria. The curves illustrate the effect of solid-fraction weighting and should not be interpreted as statistically validated probability distributions or as independent evidence of predictive accuracy.

The proposed methodological enhancements aim to improve the precision of porosity forecasting, ultimately enabling better control of casting defects, and increasing the mechanical reliability of steel components.

In metallurgical practice, porosity is one of the key defects that significantly reduces the mechanical reliability and operational performance of cast components. The primary cause of porosity in steel castings is shrinkage of the liquid phase during cooling and solidification. The Thercast NxT 3.0 software package allows simulation of temperature gradient distribution, cooling rates, and identification of potential shrinkage zones based on the Niyama criterion at the casting stage [23].

Published critical values near 0.9–1.0 K^1/2^·min^1/2^·cm^−1^ refer to the conventional Niyama criterion and are cited as literature guidance rather than directly transferred to the present model. In this study, G is expressed in K·mm^−1^, |dT/dt| in K·s^−1^, and N_y_ in K^1/2^·s^1/2^·mm^−1^. The value 0.0005 is treated as a case-specific contour level in the investigated model, not as a universal critical threshold [24].

The numerical analysis was performed in THERCAST Nxt 2.1 using the three-dimensional geometry of the investigated casting and feeding system. An unstructured mesh with a characteristic element size of 1.5–3.0 mm was used, with local refinement to 0.8–1.0 mm in thermal-concentration and defect-prone regions. The transient solution employed an adaptive time step of 0.02–0.10 s with energy-norm convergence control. Metal–mold heat transfer was represented by an effective interfacial coefficient of 400–900 W·m^−2^·K^−1^, while free surfaces were subjected to convective–radiative heat exchange. Phase change was calculated with latent-heat treatment, and temperature-dependent material properties were taken from the THERCAST material database for AISI 1045 and AISI 316L.

## 3. Results and Discussion

**Figure 3:**
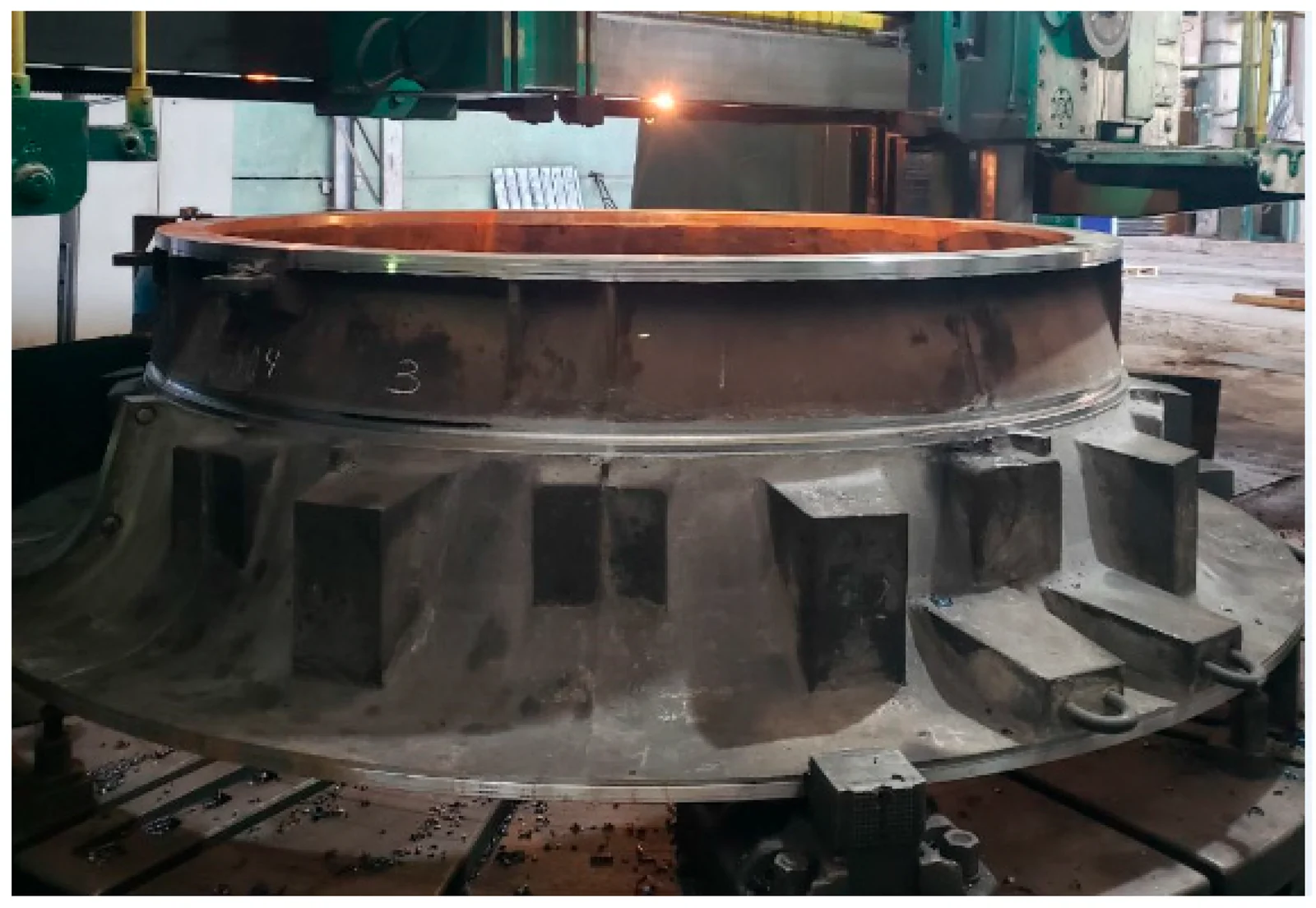
Mining ventilator cast.

The proposed approach was assessed for a mining-ventilator casting using industrial non-destructive ultrasonic inspection (Figure 3). Ultrasonic scanning identified internal reflectors in a region previously classified by the simulation as defect-prone. Targeted cutting and surface removal subsequently confirmed internal cavities in the indicated region. Because the retained inspection records do not provide a statistically complete pore-size distribution, the experimental comparison is used for spatial localization rather than for quantitative micro-porosity metrology.

**Figure 4:**
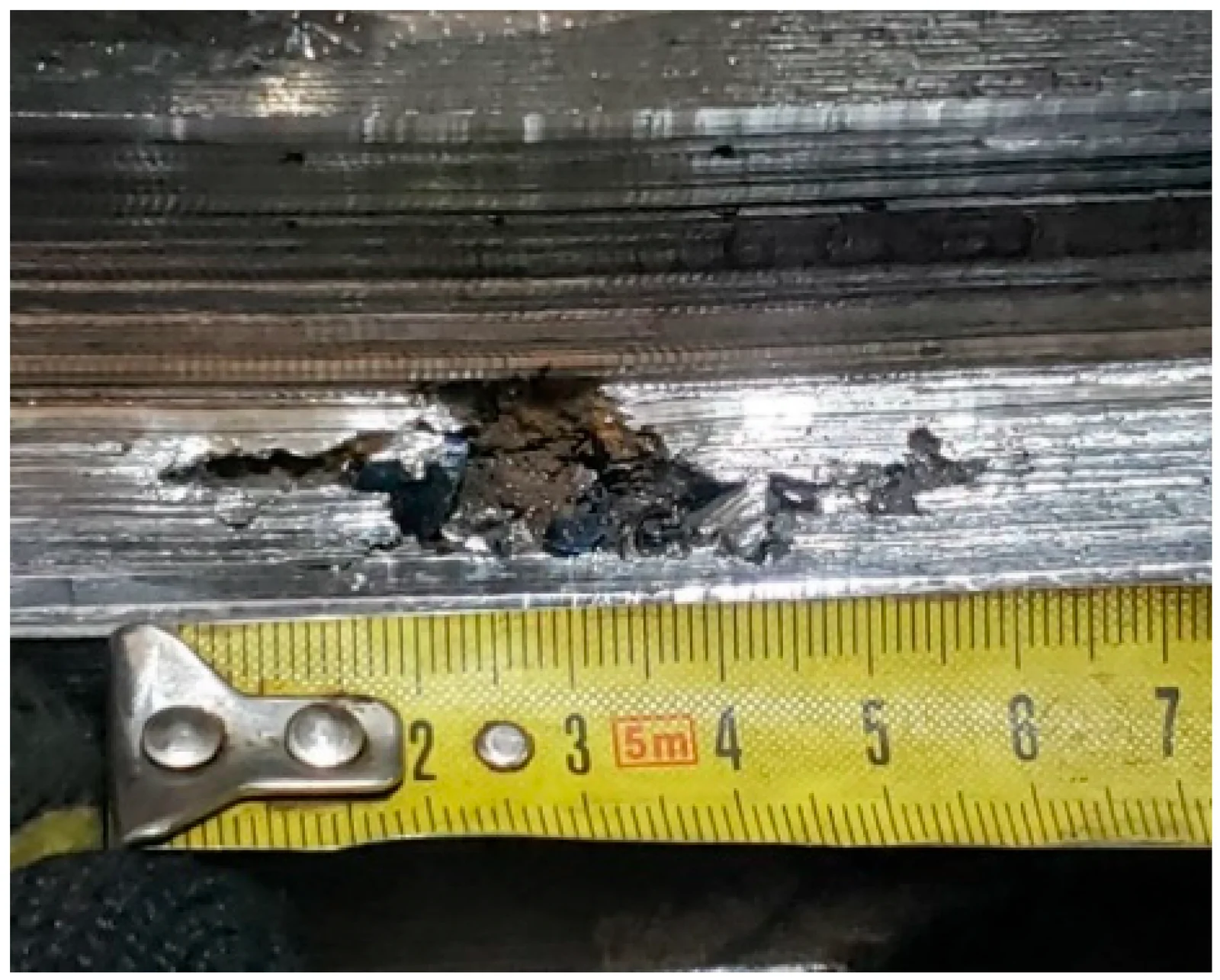
Close-up of a local cavity cluster exposed after sectioning the ultrasonically indicated area.

After cutting off the metal alloy in the defective areas, hidden gas porosity was detected (Figure 4). The steel casting was examined following the detection of subsurface defects identified via ultrasonic inspection. Subsequent cutting and removal of the upper layer revealed internal porosity extending along multiple tracks of approximately 30–31 cm. This preliminary assessment offers a perspective on the observed porosity, discussing likely formation mechanisms, its influence on structural integrity, and recommended avenues for further in-depth analysis by means of finite element analysis.

As mentioned above, casting defects can arise from numerous factors, including inadequate feeding during solidification, improper mold design, thermal gradients, or material composition of inconsistencies. Even minor subsurface porosity may pose a serious threat to mechanical reliability, especially in load-bearing applications. The defect under examination was discovered through ultrasonic testing and visually confirmed after the removal of the casting’s upper layer, confirming internal porosity over a length of roughly 10 cm in the photographed region (Figure 4).

**Figure 5:**
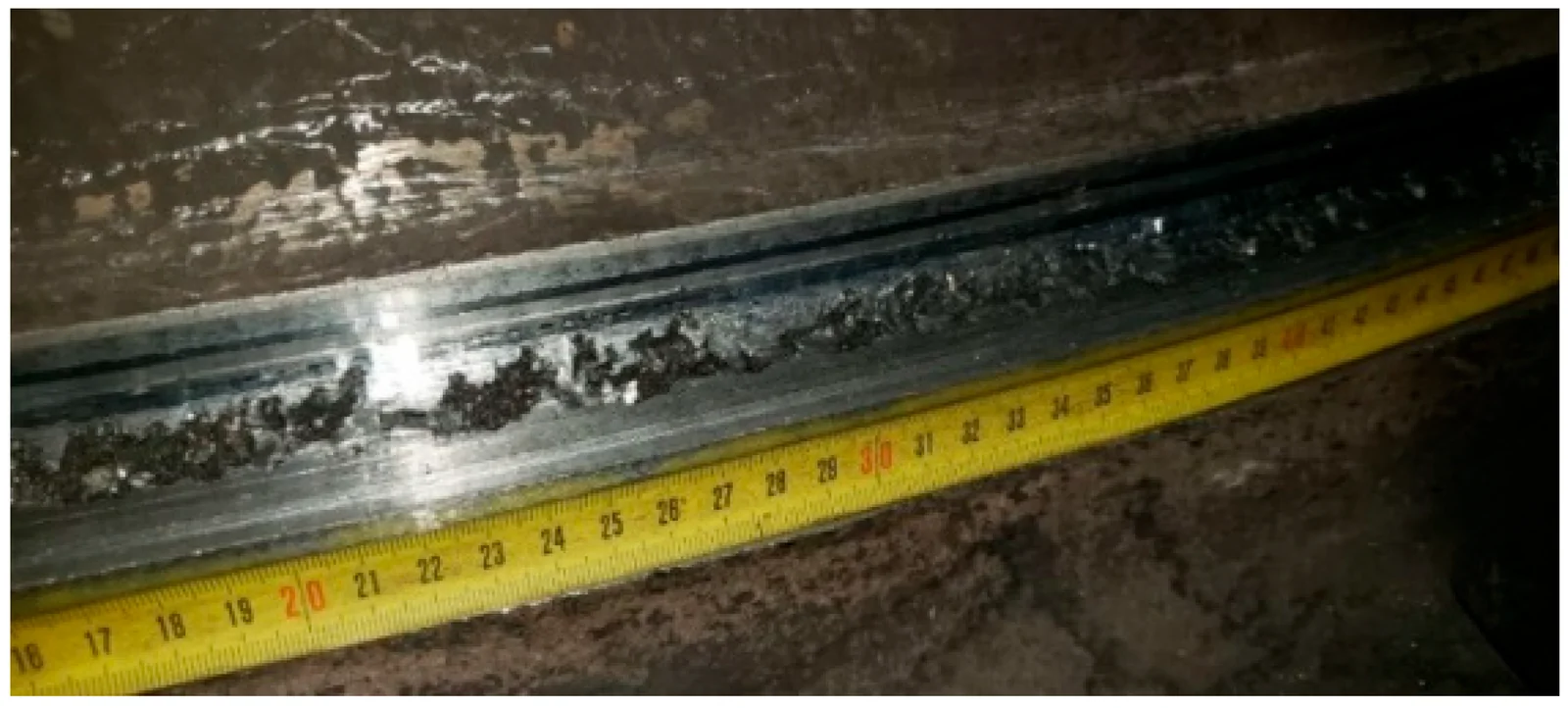
Overview of the extended internal-defect track along the inspected casting section.

The photograph (Figure 5) shows clusters of uneven cavities that appear interconnected, suggesting they may have formed during the late stages of solidification, possibly due to inadequate feeding or entrapped gas. The pores exhibit varying diameters, with some merging to create irregular channels.

These observations strongly support the idea that the detected defects correspond to true subsurface porosity as initially flagged by ultrasonic scanning. Common causes include shrinkage porosity in the event that the solidification front advanced unevenly or riser feed paths were insufficient, as well as gas porosity, evolving gases that remain dissolved in the molten metal, which precipitate out during cooling and become trapped and form spherical or elongated voids.

Thus, the Niyama simulation of the cast with known defects can facilitate verification of the modified Niyama criterion.

For the finite element analysis in Thercast, the simulation has been provided for the denoted cast to validate the existing defects by the proposed prediction method. The research focused on two grades of steel used in casting: AISI 1045 (medium-carbon steel) and AISI 316L (austenitic stainless steel).

AISI 316L is an austenitic stainless-steel alloy known for its excellent corrosion resistance, particularly in chloride-rich environments, and is widely used in marine, medical, and chemical processing applications. In the context of ingot casting, 316L presents favorable casting characteristics due to its relatively low carbon content (<0.03%), which minimizes carbide precipitation during solidification and subsequent cooling. This enhances intergranular corrosion resistance and ensures structural homogeneity. However, casting 316L still poses challenges related to hot tearing, macrosegregation, and the potential formation of shrinkage porosity due to its high alloying content (notably molybdenum and chromium), which influences the solidification range and increases susceptibility to interdendritic shrinkage.

During solidification, AISI 316L undergoes a peritectic reaction, with the liquid transforming into delta ferrite and then austenite, depending on cooling rate and composition. This dual-phase evolution can affect feeding ability and porosity formation. The wide freezing range of 316L increases the mushy zone thickness, reducing feeding efficiency and promoting porosity if not properly controlled. Simulation tools like Thercast or ProCAST are often used to evaluate thermal gradients and predict high-risk zones for shrinkage. To improve ingot quality, process parameters such as mold design, cooling rate, and riser geometry must be carefully optimized. Post-casting treatments like hot isostatic pressing (HIP) are also frequently employed to close internal voids and enhance the integrity of large 316L castings.

The chemical composition of these steels significantly affects their solidification behavior and porosity formation. AISI 1045 contains approximately 0.45% C, 0.75% Mn, and 0.03% S, while AISI 316L includes 16–18% Cr, 10–14% Ni, and 2–3% Mo, which enhances corrosion resistance but affects the solidification of shrinkage characteristics.

**Figure 6:**
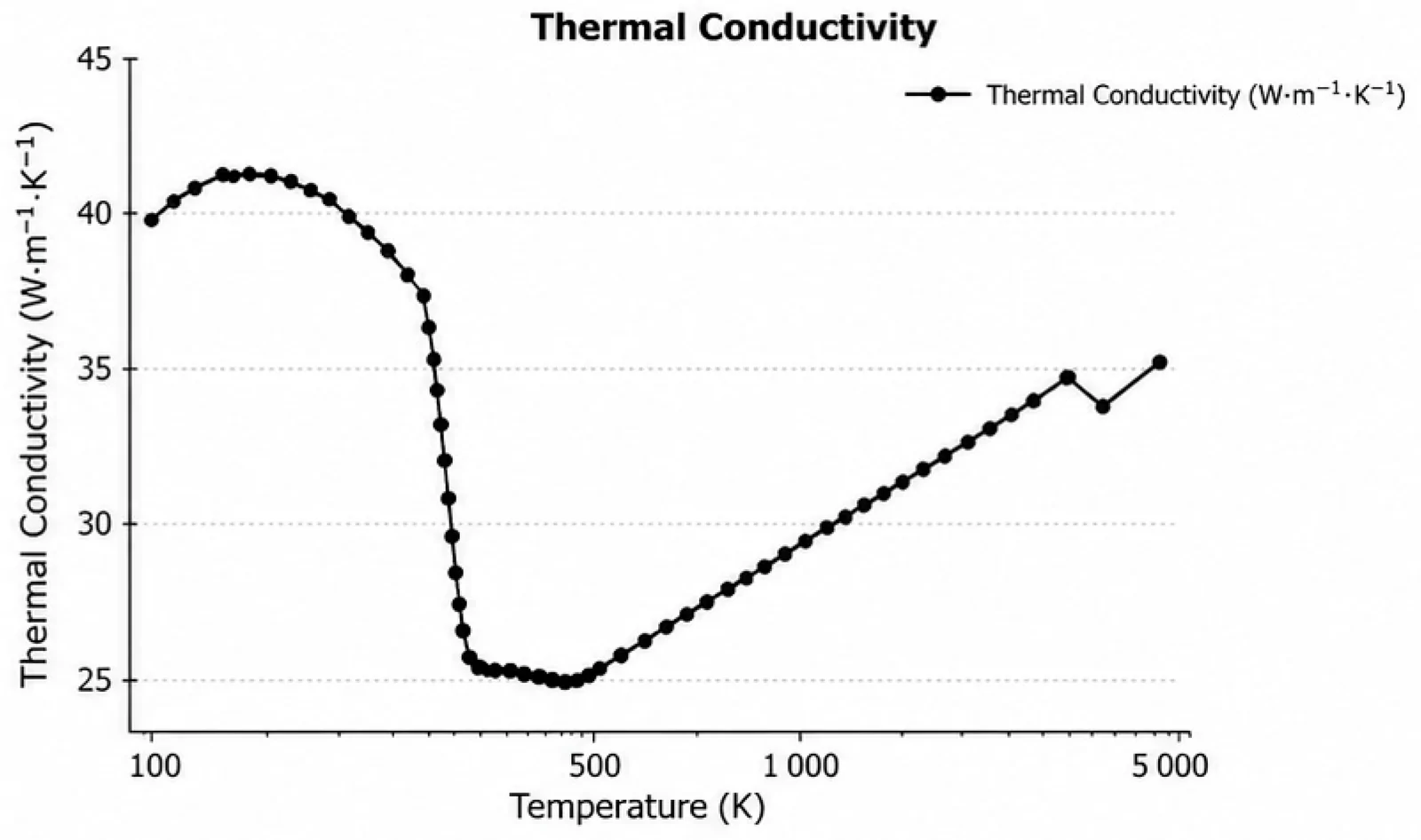
Thermal conductivity of cast steel.

The thermal properties of these steels were considered in the simulation. The thermal conductivity of AISI 1045 was represented in the model by a reference value of 50 W/m·K, while AISI 316L was represented by a lower reference value of 25 W/m·K (Figure 6), influencing heat dissipation and cooling rates. The solidification temperature range was determined to be 1450–1500 °C for AISI 1045 and 1370–1400 °C for AISI 316L, which affects micro-porosity development [17].

In computational metallurgy, the numerical simulation of casting processes follows three essential stages: pre-processing, which involves geometry modeling and computational grid generation; processing, where the solver performs the necessary calculations; and post-processing, which focuses on analyzing and interpreting results. These steps are crucial for optimizing ingot production and ensuring high-quality steel manufacturing.

The influence of porosity on tensile strength was evaluated using the analytical relation presented in Equation (4) and compared with published trends for cast alloys. The 30% value cited in the original wording should be understood as a possible upper-range, material-dependent reduction rather than as a universal result of a new tensile-test series in this study. The principal experimentally verified result of the present work is the spatial correspondence between the simulated defect-prone region and the cavities revealed by ultrasonic inspection and sectioning.

Thercast, a finite element-based simulation tool, provides detailed modeling of porosity formation in carbon steel castings by integrating solidification physics with thermal and metallurgical parameters. One of its key strengths lies in simulating heat transfer and phase change dynamics during cooling, which are critical to understanding shrinkage porosity formation. By solving transient heat conduction equations with latent heat release and temperature-dependent material properties, Thercast accurately captures the evolution of temperature gradients and solidification of front progression. This thermal analysis is fundamental to identifying “hot spots” or thermal centers within the casting, where the risk of shrinkage porosity is highest due to delayed solidification and insufficient feeding.

In addition to thermal modeling, Thercast incorporates porosity prediction algorithms based on empirical and semi-empirical criteria, such as the Niyama criterion. Regions with low Niyama values indicate conditions that promote shrinkage porosity due to inadequate feeding pressure during the final stages of solidification. By mapping these critical areas, the software helps visualize potential defect zones and test alternative gating or risering configurations without physical prototyping. Furthermore, Thercast can simulate the impact of casting geometry, alloy composition, and mold material properties on porosity development, providing a comprehensive platform for virtual casting optimization.

Furthermore, Thercast can simulate the effects of casting geometry, alloy composition, and mold design on porosity evolution. It enables users to optimize riser placement, feeding paths, and cooling rates by evaluating the shrinkage of evolution over time. For carbon steels, where the solidification range is relatively narrow, the accuracy of thermal modeling is crucial. The simulation can also consider latent heat of fusion, temperature-dependent material properties, and phase transformation kinetics, such as the austenite–ferrite transition. Altogether, Thercast provides a predictive and visual analysis of porosity risks, supporting decision making in casting process design and helping reduce costly defects in carbon steel components.

Particularly critical is porosity located in the core of the casting, where operational stress amplitudes are the highest. Pore size and internal stress concentration are critical factors influencing the mechanical performance of cast steels. According to Lamé’s theory of stress distribution around cavities in elastic solids, spherical pores act as local stress concentrators, where the circumferential (hoop) stress around the pore is amplified relative to the nominal applied stress. The theoretical maximum hoop stress (σ_θ_) around a spherical void can be estimated as [25]:

(3) $$\sigma_{\theta }=\sigma_{app}(1+\frac{2a^{3}}{r^{3}}),$$

where $\sigma_{app}$ - is the applied stress, *a* is the radius of the pore, and *r* is the radial distance from the center of the pore. At the pore surface (r = a), Equation (3) gives σ_θ_/σ_app_ = 1 + 2a^3^/a^3^ = 3; thus, the local stress concentration factor is three within the assumptions of the idealized spherical-cavity model. Consequently, even moderate external stresses can result in localized overstressing near pores, especially when the material’s ductility is low, or the loading rate is high.

In carbon and stainless steels such as AISI 316L, when pores exceed approximately 100 μm in diameter and are subjected to local stress levels above ~150 MPa, the surrounding matrix material becomes highly vulnerable to failure initiation. This is particularly relevant under dynamic or tensile loading, where the localized stress intensity may exceed the critical threshold for brittle fracture. Intergranular failure becomes prominent because the presence of porosity weakens grain boundary cohesion and enables crack nucleation along these interfaces. The pore effectively acts as a crack-like defect with a high stress intensity factor, pushing it closer to the material’s fracture toughness limit.

Such fracture behavior is exacerbated by poor microstructural continuity around the pore, where shrinkage defects often correlate with regions of solidification segregation. Alloying elements like sulfur and phosphorus may accumulate at grain boundaries, further reducing cohesion and promoting brittle fracture. The problem becomes critical in components that must sustain cyclic or high-magnitude loads, such as pressure vessels or structural steel castings. Therefore, simulations using Lamé theory, in conjunction with fracture mechanics models, are essential for predicting failure probability based on pore geometry, location, and loading conditions. This insight informs quality control thresholds for acceptable pore sizes and justifies post-casting treatments like hot isostatic pressing (HIP) to eliminate critical voids.

**Figure 7:**
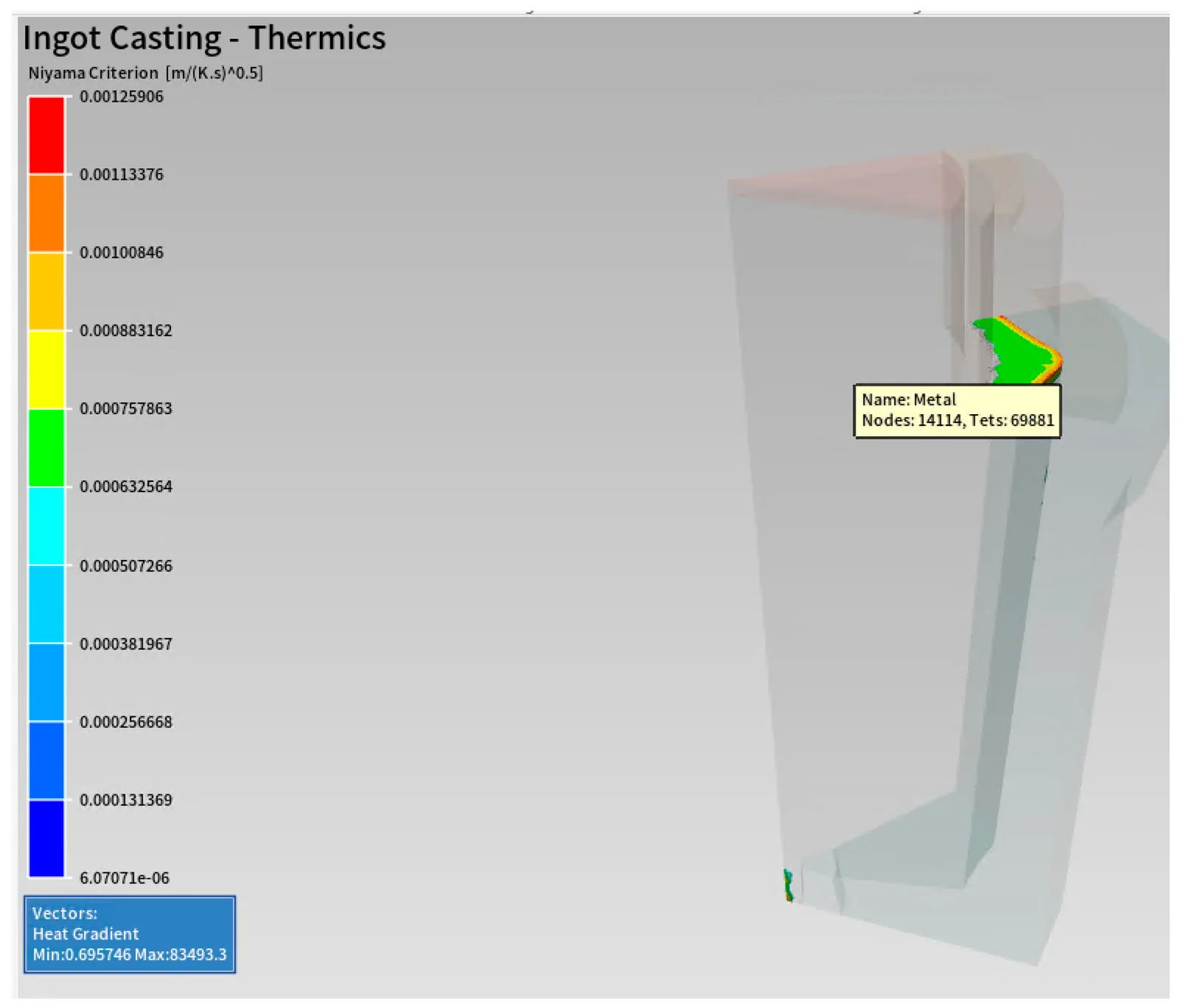
Porosity distribution simulated by Thercast.

**Figure 8:**
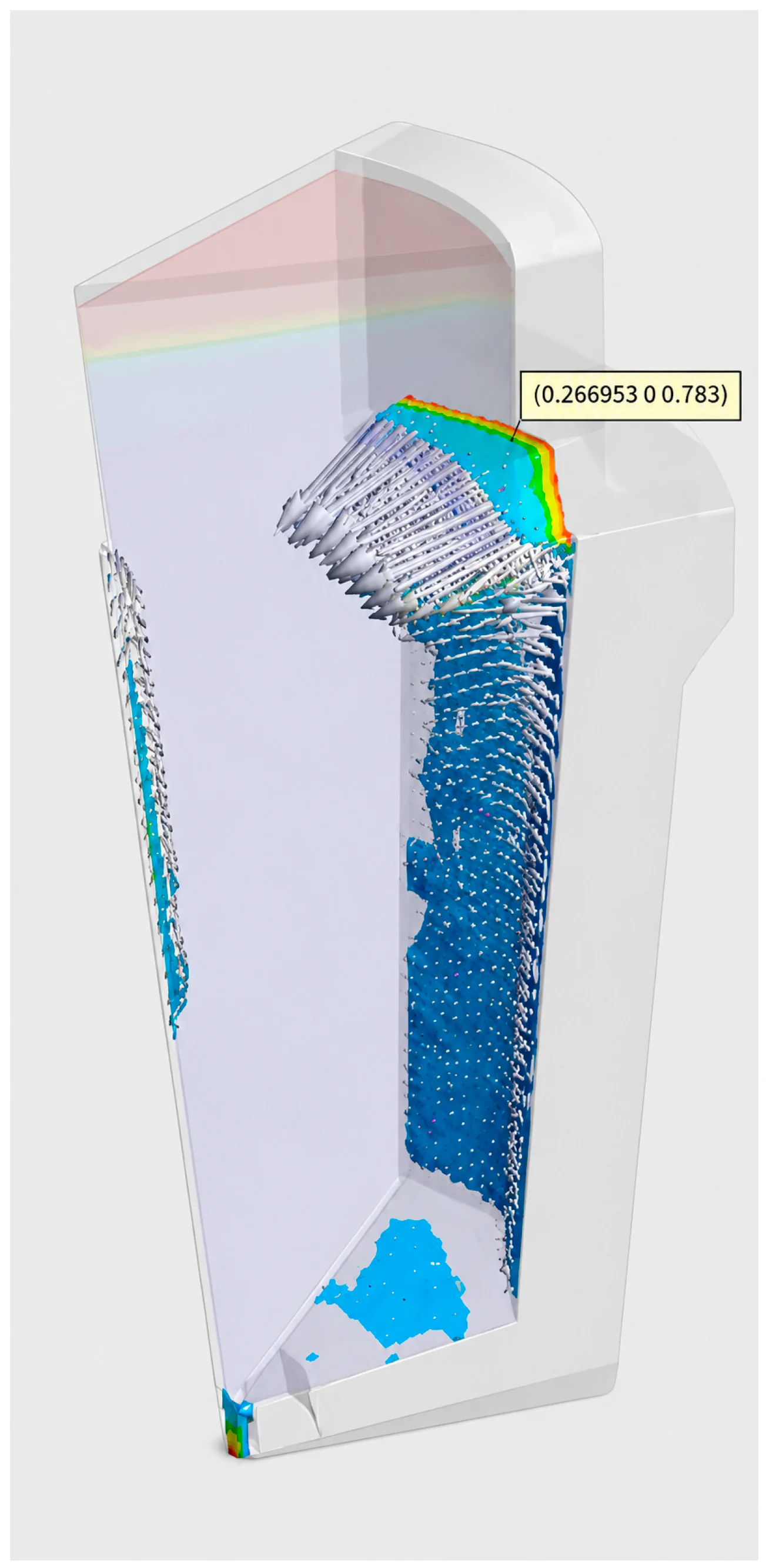
Cast solidification direction.

The porosity distributions obtained from THERCAST simulations (Figure 7 and Figure 8) showed that AISI 316L was more susceptible to porosity localization because of its lower reference thermal conductivity and wider solidification interval. The conventional Niyama map exhibited a pronounced low-value region near the case-specific contour level of 0.0005 K^1/2^·s^1/2^·mm^−1^. The modified indicator improved the spatial discrimination of this region through the explicit solid-fraction term; viscosity, composition, and shrinkage affect the result through the underlying THERCAST material and solidification model.

**Figure 9:**
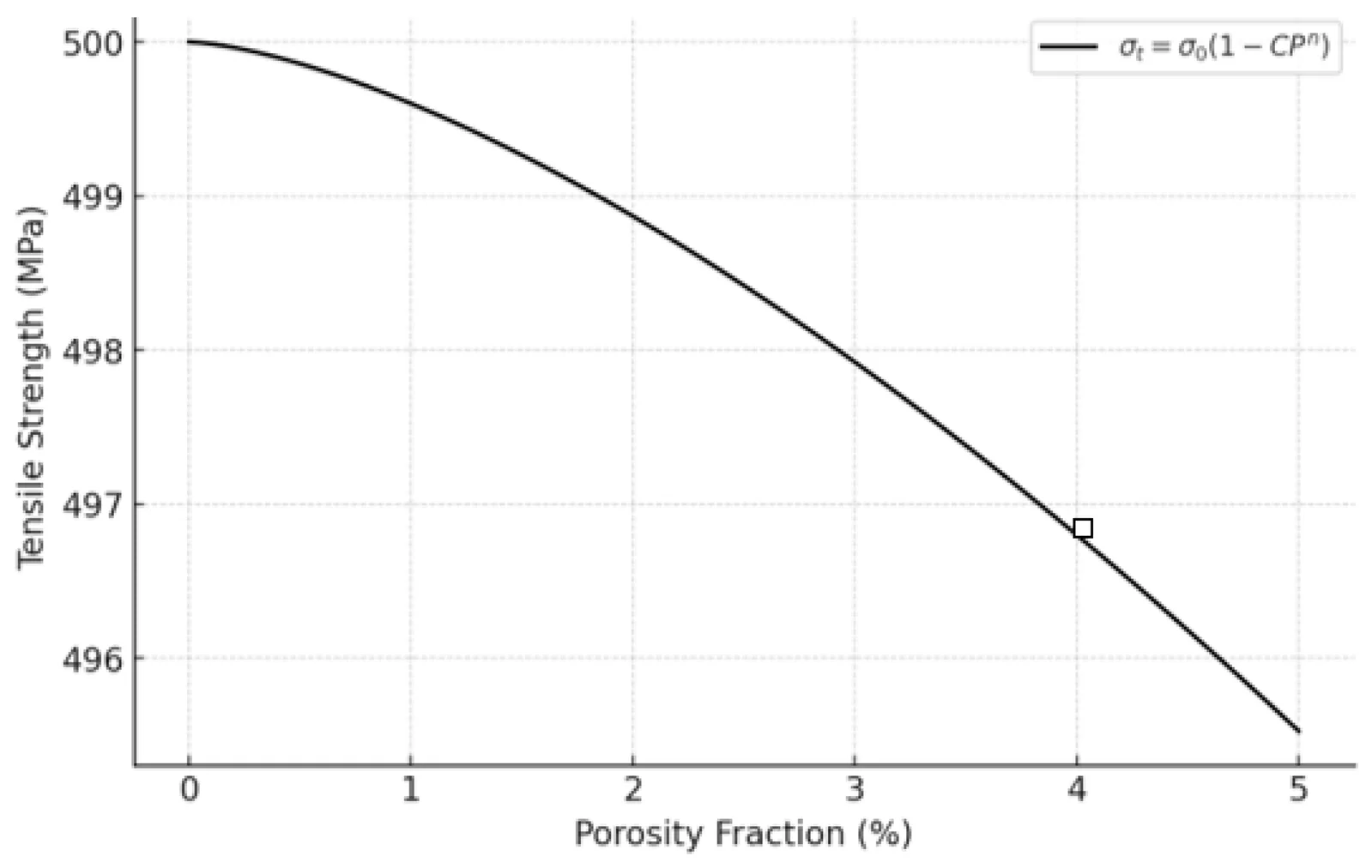
Illustrative relation between normalized tensile strength and porosity fraction according to Equation (4).

Mathematical relations describing porosity impact on mechanical properties were derived using fracture mechanics principles. The tensile strength *σ_t_* was related to porosity fraction *P* by:

(4) $$\sigma_{t}=\sigma_{0}(1-{CP}^{n}),$$

where σ_0_ is the tensile strength of the defect-free material, P is the porosity fraction expressed as a decimal, and C and n are empirical, material-dependent coefficients. Accordingly, Equation (4) and Figure 9 illustrate the expected trend and do not constitute a universal calibration for AISI 1045 or AISI 316L.

The thermal gradient *G* plays a decisive role in the morphology and stability of the solidification front during the casting of steel components. A high thermal gradient promotes directional solidification, enabling the molten metal to feed the contracting solid phase more effectively, thereby minimizing shrinkage porosity. Conversely, a low thermal gradient leads to the formation of isolated solid zones and interdendritic regions that are poorly fed, increasing the likelihood of shrinkage voids. Numerical simulations in Thercast demonstrate the fact that regions where *G* < 10 K/mm are more susceptible to the formation of microporosity, particularly when combined with moderate to high cooling rates.

The Niyama criterion quantifies the joint influence of thermal gradient and cooling rate on porosity risk. When the thermal gradient decreases while the cooling rate remains relatively high, the Niyama value decreases and the susceptibility to restricted feeding increases. In the investigated casting, the low-Niyama region corresponded spatially to the defect-bearing area identified by ultrasonic inspection and subsequent sectioning. The literature threshold of approximately 1.0 K^1/2^·min^1/2^·cm^−1^ is not directly compared with the case-specific contour values without unit conversion and calibration.

These results indicate that solid-fraction weighting improves the spatial discrimination of defect-prone regions in the investigated casting, while the conventional and modified indicators retain the same physical interpretation: lower values correspond to less favorable feeding conditions.

The spatial localization capability of Niyama-based porosity prediction was assessed through complementary experimental techniques, including ultrasonic testing and metallographic sectioning. In particular, areas predicted by simulation to have subcritical Niyama values were found to correlate strongly with zones of high defect density in real castings. Ultrasonic scanning revealed increased echo loss and scatter in these zones, indicating internal voids or micropores, while polished metallographic cross-sections confirmed the presence of shrinkage cavities and interdendritic porosity. These findings substantiate the physical relevance of the Niyama criterion and highlight its predictive power in practical casting scenarios. Furthermore, this synergy between simulation and experiment emphasizes the importance of simulation-driven optimization in modern foundry practice.

From a process engineering perspective, the ability to control and optimize thermal gradients during solidification is vital for minimizing porosity and achieving reliable mechanical performance. This can be achieved by adjusting mold geometry, implementing directional solidification strategies, or enhancing heat extraction rates through mold chilling or thermal insulation. For instance, increasing the thermal gradient via improved heat sink placement or mold material selection can maintain Niyama values above the critical threshold throughout the ingot. Additionally, by reducing thermal bottlenecks and ensuring progressive solidification from hot spots toward the risers, feeding pressure is preserved, thereby minimizing the nucleation of shrinkage cavities. Ultimately, the integration of Niyama-based simulations into casting design enables predictive defect control, material property enhancement, and reduction in costly trial-and-error in casting development [19].

**Figure 10:**
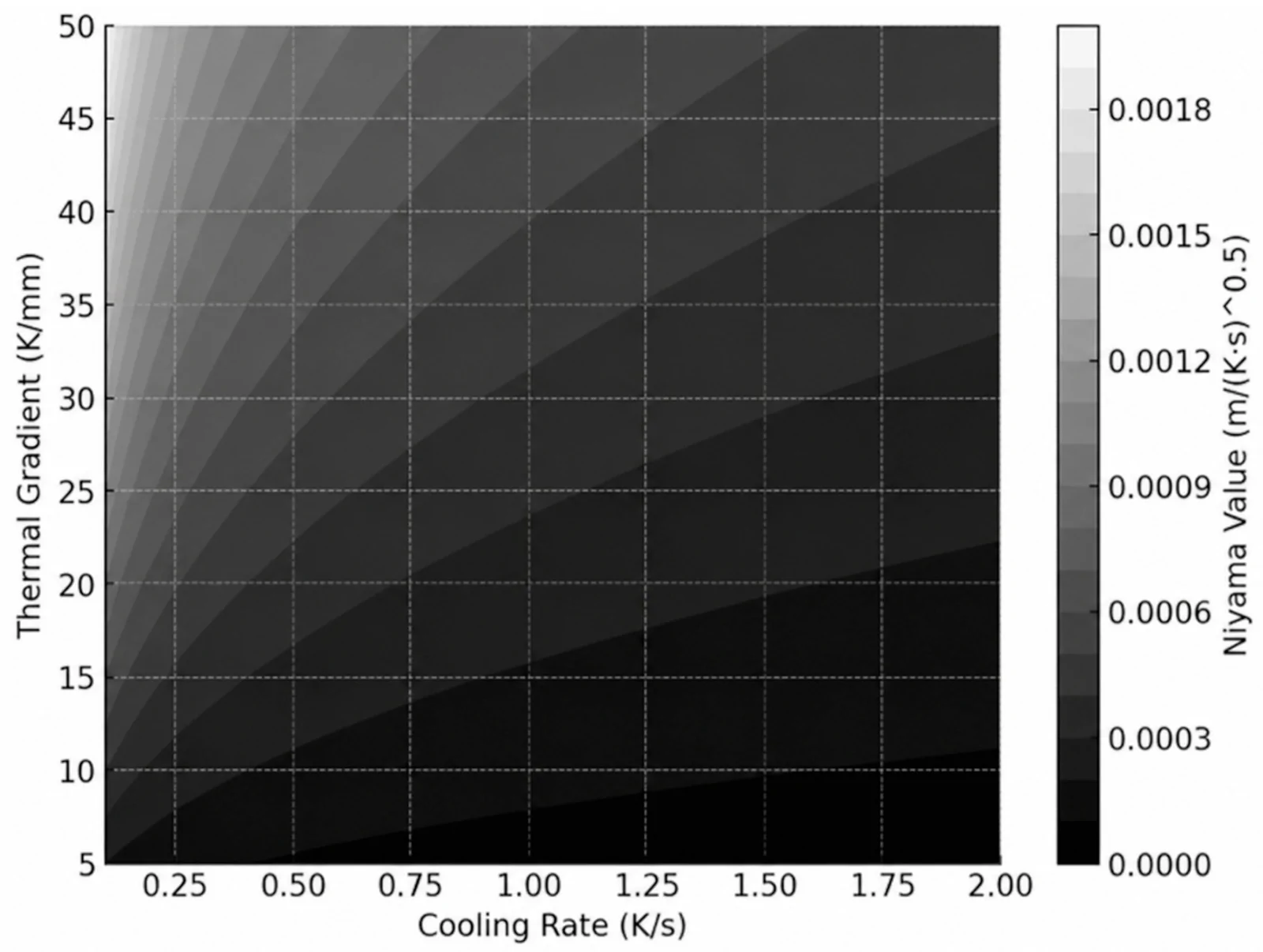
Heat transfer gradient influence in Niyama criterion.

A detailed analysis of the thermal-gradient and cooling-rate contributions to the Niyama indicator is presented in Figure 10. The plotted values of the case-specific indicator range from 0 to 0.002 K^1/2^·s^1/2^·mm^−1^. For a fixed thermal gradient, a higher cooling rate decreases N_y_; in an actual casting, however, G and |dT/dt| vary simultaneously and must be interpreted through the complete thermal field.

**Figure 11:**
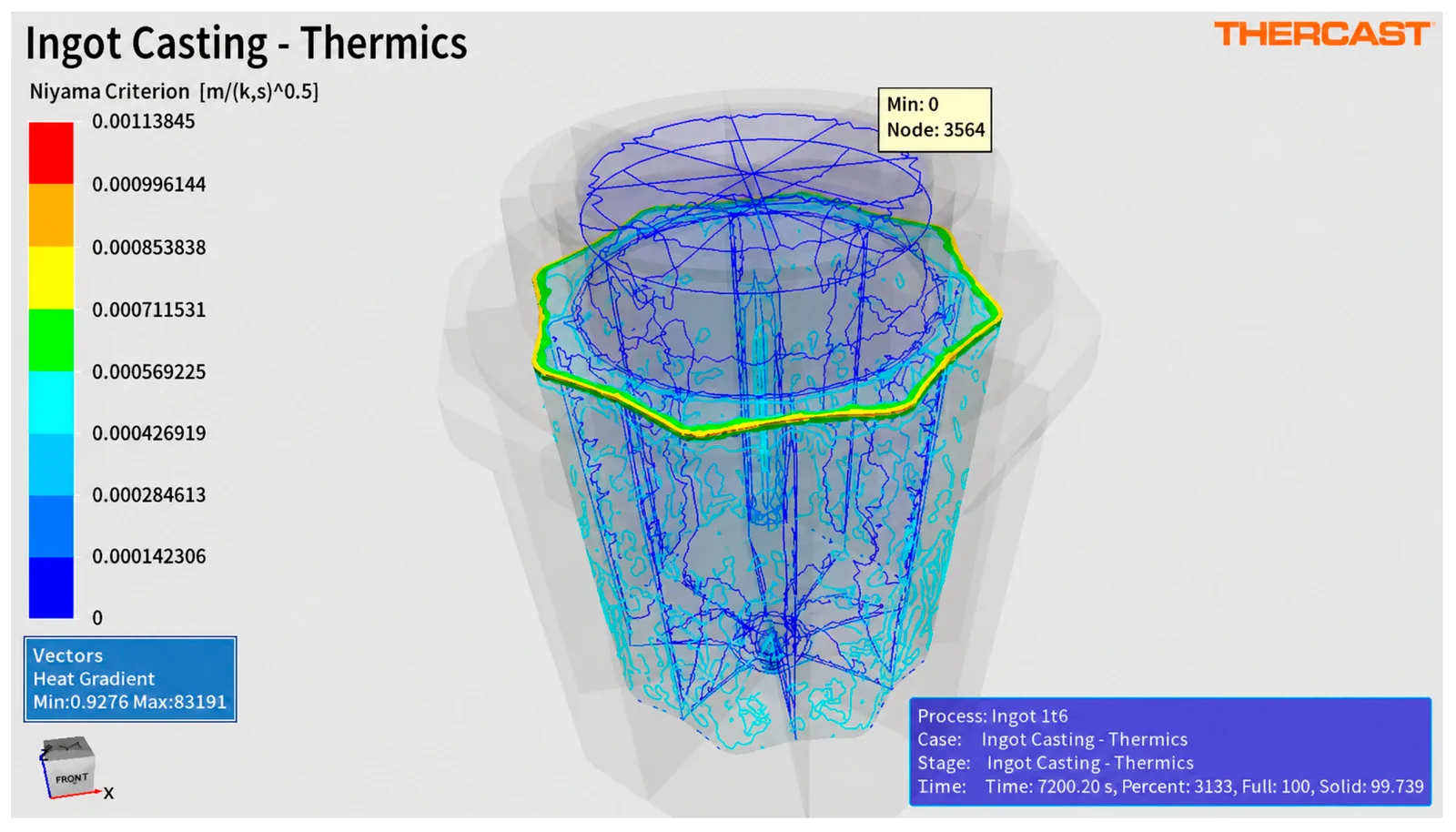
Simulation of Niyama criterion distribution.

For the evaluated cooling-condition variant, reducing the characteristic cooling rate to approximately 1.25 K/s decreased the extent of the simulated cross-sectional region classified as porosity-prone relative to the baseline case (Figure 11). The comparison therefore demonstrates a relative change in the predicted risk-zone area and should not be interpreted as a directly measured reduction in pore volume.

The simulated porosity-prone region showed substantial spatial coincidence with the defect-bearing region identified by ultrasonic inspection in the inspected section. This comparison is used as spatial validation of defect localization and is not interpreted as a statistical classification accuracy, sensitivity, or specificity metric. Shrinkage-related porosity is associated with lower, not higher, Niyama values. Adjusting riser design, pouring temperature, or local cooling conditions may therefore reduce internal defects by improving directional feeding.

## 4. Conclusions

The research successfully demonstrates the effectiveness of Thercast finite element analysis in predicting and mitigating porosity defects in steel castings. By supplementing the Niyama criterion with explicit solid-fraction weighting, while retaining alloy-dependent thermophysical effects in the THERCAST solver, the spatial discrimination of porosity-prone regions was improved for the investigated casting. The numerical results indicate that decreasing the characteristic cooling rate to 1.25 K/s reduced the extent of the simulated porosity-prone region in the investigated casting configuration. The low-value region of the modified indicator showed substantial spatial coincidence with the defect-bearing region in the inspected section; the contour level of 0.0005 K^1/2^·s^1/2^·mm^−1^ is treated as case-specific rather than universal. The integration of numerical simulations with experimental validation confirms that a higher thermal gradient and optimized cooling rates contribute to defect reduction. Furthermore, the analytical porosity–strength relation confirms a material-dependent decrease in tensile strength as porosity increases; the previously cited 30% value is therefore interpreted as an upper-range estimate rather than a universal experimental result. These findings provide valuable insights for foundries aiming to enhance casting quality and reliability while advancing metallurgical production methodologies.

## Data Availability

The original contributions presented in this study are included in the article. Further inquiries can be directed to the corresponding author.
